# A method for high-throughput gene expression signature analysis

**DOI:** 10.1186/gb-2006-7-7-r61

**Published:** 2006-07-19

**Authors:** David Peck, Emily D Crawford, Kenneth N Ross, Kimberly Stegmaier, Todd R Golub, Justin Lamb

**Affiliations:** 1 Broad Institute of MIT and Harvard, Cambridge, Massachusetts 02142, USA; 2 Dana-Farber Cancer Institute, Boston, Massachusetts 02115, USA; 3 Howard Hughes Medical Institute, Dana-Farber Cancer Institute, Boston, Massachusetts 02115, USA

## Abstract

Genome-wide transcriptional profiling has shown that different biologic states (for instance, disease and response to pharmacologic manipulation) can be recognized by the expression pattern of relatively small numbers of genes. However, the lack of a practical and cost-effective technology for detection of these gene expression 'signatures' in large numbers of samples has severely limited their exploitation in important medical and pharmaceutical discovery applications. Here, we describe a solution based on the combination of ligation-mediated amplification with an optically addressed microsphere and flow cytometric detection system.

## Abstract 2

A new method for cost-effective high-throughput gene expression signature analysis is described.

## Background

Gene expression signatures comprised of tens of genes have been found to be predictive of disease type and patient response to therapy, and have been informative in countless experiments exploring biological mechanism (for example [1-4]). High-density DNA microarrays therefore represent the method of choice for unbiased transcriptome analysis and represent an excellent route to signature discovery. However, gene expression signatures with diagnostic potential must be validated in large cohorts of patients, in whom measuring the entire transcriptome is neither necessary nor desirable. Perhaps more important is that the ability to describe cellular states in terms of a gene expression signature raises the possibility of performing high-throughput, small-molecule screens using a signature of interest as the read out. However, for this to be practical one would need to be able to screen thousands of compounds per day at a cost dramatically below that of conventional microarrays.

**Figure 1:**
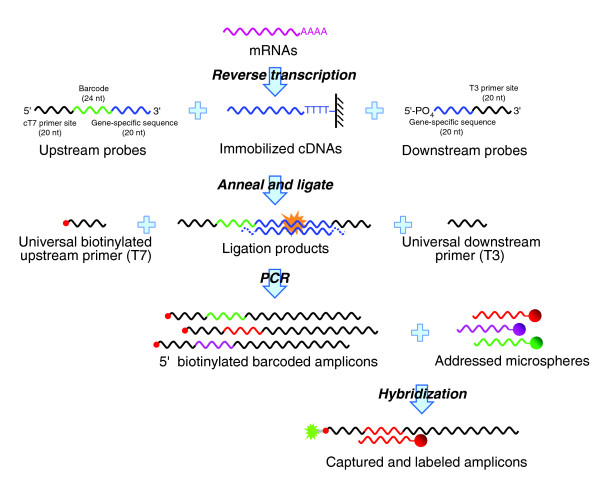
Method overview. Transcripts are captured on immobilized poly-dT and reverse transcribed. Two oligonucleotide probes are designed against each transcript of interest. The upstream probes contain 20 nt complementary to a universal primer (T7) site, one of 100 different 24 nt barcode sequences, and a 20 nt sequence complementary to the 3'-end of the corresponding first-strand cDNA. The downstream probes are 5'-phosphorylated and contain a 20 nt sequence contiguous with the gene-specific fragment of the upstream probe and a 20 nt universal primer (T3) site. Probes are annealed to their targets, free probes removed, and juxtaposed probes joined by the action of ligase to yield synthetic 104 nt amplification templates. PCR is performed with T3 and 5'-biotinylated T7 primers. Biotinylated barcoded amplicons are hybridized against a pool of 100 sets of optically addressed microspheres each expressing capture probes complementary to one of the barcodes, and incubated with streptavidin-phycoerythrin to label biotin moieties fluorescently. Captured labeled amplicons are quantified and beads decoded by flow cytometry. nt nucleotides.

We therefore developed a simple, flexible, cost-effective, and high-throughput gene expression signature analysis solution tailored for the measurement of up to 100 transcripts in many thousands of samples by combining multiplex ligation-mediated amplication [5-7] with the Luminex FlexMAP (Luminex, Austin, TX, USA) optically addressed and barcoded microsphere and flow cytometric detection system, that we together refer to as LMF (Figure 1) [8]. Here, we detail the LMF method and report on its overall performance.

## Results and Discussion

To test the LMF method a 90-gene expression signature was derived from an unbiased genome-wide transcriptional analysis of a cell culture model of hematopoietic differentiation. Total RNA was isolated from HL60 cells following treatment with tretinoin (all-*trans *retinoic acid) or vehicle (dimethyl sulfoxide [DMSO]) alone, amplified and labeled by *in vitro *transcription (IVT), and hybridized to Affymetrix GeneChip microarrays (Affymetrix Inc., Santa Clara, CA, USA). Features reporting above threshold were binned into three groups of equal size on the basis of expression level (low = 20-60 units; medium = 60-125 units; and high = >125 units). Ten transcripts exhibiting low (1.5-2.5×), moderate (3-4.5×), and high (>5×) differential expression between the two conditions were then selected from each bin, populating a matrix of nine classes (Additional data file 1), thereby spanning the range of differential expression likely to be encountered in a typical signature analysis experiment.

**Figure 2:**
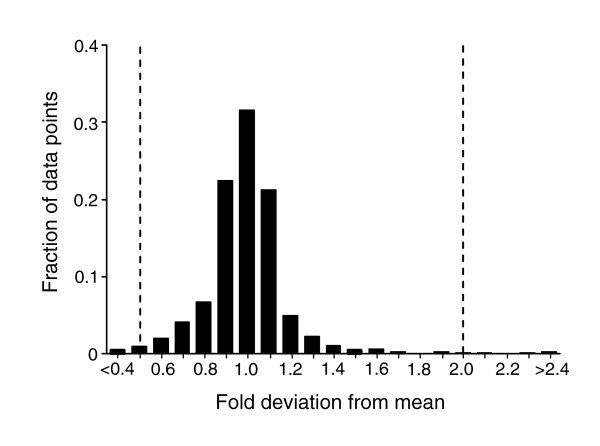
Reproducibility of the method. Mean expression levels for each transcript under each condition were computed and the deviation of each individual data point from its corresponding mean was recorded. A histogram of the fraction of data points in each of 12 bins of fold deviation values is shown. This plot represents 1,800 data points (two conditions × 90 transcripts × 10 replicates).

Probe pairs were designed against each of the 90 transcripts (Additional data file 2) and tested against 10 aliquots of the tretinoin-treated and vehicle-treated HL60 RNA to provide a measure of the reproducibility of LMF. Replicate measurements were highly correlated, with 97.9% of data points falling within twofold of their corresponding means (Figure 2). Much of the variability was explained by a single transcript, accounting for 34% of the data points outside this range. The overall reproducibility of the assay was therefore extremely high.

**Figure 3:**
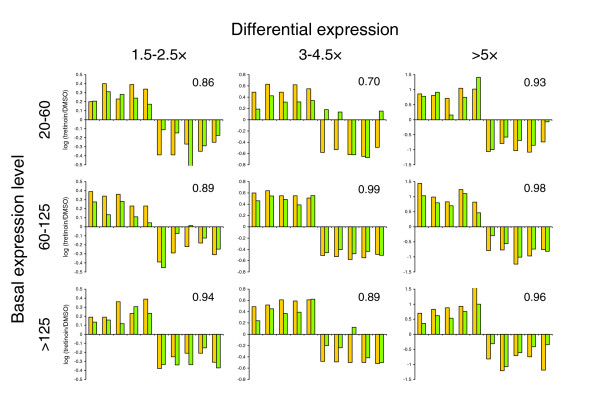
Performance in a representative gene space. Total RNA from HL60 cells treated with tretinoin or vehicle (DMSO) alone were analyzed by LMF in the space of 90 transcripts selected from microarray analysis of the same material. Plots depict log ratios of expression levels (tretinoin/DMSO) reported by both platforms for each transcript, in each of nine classes. Correlation coefficients of the log ratios between platforms within each class are shown. Yellow bars represent microarray findings and green bars LMF findings. Ratios were computed on the means of three parallel hybridizations of the pooled product from three amplification and labeling reactions (microarray) or 10 parallel amplification and hybridization procedures (LMF) for each condition. Basal expression categories are 20-60 (low), 60-125 (moderate), and >125 (high). Differential expression categories are 1.5-2.5× (low), 3-4.5× (moderate), and >5× (high). DMSO, dimethyl sulfoxide.

We next considered the extent to which LMF could recapitulate the gene expression signature discovered with microarrays. Estimates of the extent of differential expression reported by both solutions were very similar, even in the low basal and low differential expression classes (Figure 3). Five probe pairs produced gross errors, which is in accordance with our typical first-pass probe failure rate of 5%. All failures can generally be remedied by probe redesign. The overall correlation of log ratios between the platforms across all 90 transcripts was 0.924, demonstrating that for the vast majority of transcripts the LMF method faithfully recovered the microarray-defined changes. A repeat of this entire LMF analysis on two additional occasions yielded similar results. The coefficient of variation of mean expression level for each of the 90 features across all three independent evaluations had a mean of 13.8% (range 1.1-49.8%) indicating high stability of the platform.

The most important attribute of a signature analysis technology is its ability to discriminate between biologic states. To test this, we used LMF to collect data for our 90 gene feature set from 94 microtiter well cultures of HL60 cells each treated with either tretinoin or vehicle. Approximately 7,500 cell equivalents were assayed from each well. LMF has been successfully applied to the analysis of clinical specimens of as few as 5,000 cell equivalents (data not shown). A *k*-nearest-neighbor (KNN) classification algorithm trained on the original 90-gene, microarray-derived expression signature delivered a classification accuracy of 100% for these samples. Classifiers built in the space of the nine previously defined expression classes (10 transcripts each) also had extremely high accuracy, ranging from 85.2% to 100% (Table 1), indicating that LMF performs well even for small signatures populated with nonabundant transcripts and with only modest differential expression. We note that the classification accuracy was high despite the tendency of LMF to underestimate the extent of differential expression relative to the Affymetrix platform (Figure 3). Indeed, KNN classifiers built and tested by leave-one-out cross-validation on positive and negative control wells from a number of actual small molecule screens performed with LMF in our laboratory also delivered impressive accuracies (30-gene androgen signature, 642 wells: 98.8% accuracy; 11-gene sarcoma signature, 766 wells: 99.6% accuracy; 21-gene adipocyte signature, 324 wells: 100% accuracy; and 11-gene neuroblastoma signature, 191 wells: 99.5% accuracy; data not shown). Taken together, these data indicate that LMF represents a highly accurate signature analysis method that is suitable for high-throughput applications.

LMF makes possible a wide variety of gene expression-based applications that have the potential to be transforming, particularly in the area of small molecule screening. High-throughput small molecule screens based on gene expression have previously been suggested, but they have been limited to the measurement of a single gene by either reporter assay or real-time PCR, or by a small signature of up to five genes using a mass spectrometric readout [9]. The ability to measure more complex (up to 100-transcript) signatures (with concomitant increases in sensitivity and robustness) in high throughput has not previously been feasible. The LMF method now makes such signature-based small molecule screening practical.

LMF should be distinguished from other bead-based mRNA expression methods. BADGE (Beads Array for the Detection of Gene Expression) combines IVT labeling with flow cytometric detection [10]. RNA-mediated annealing, selection and ligation (RASL) and DNA-mediated annealing, selection and ligation (DASL) are ligation-based methods that rely on self-assembled fibreoptic bead arrays for detection [11,12]. Although these approaches have demonstrated feasibility, the labeling costs for IVT approach US$100 (versus about $0.25 for LMF) and the costs of flow-based detection compare very favorably with those of fibreoptic bead arrays. Even the most inexpensive custom low-density microarray solutions have detection costs at least one order of magnitude greater than that of LMF and they also commonly suffer from high initial set-up charges. Furthermore, LMF has been implemented in large-scale, 384-well, plate-based screens, whereas neither BADGE or RASL/DASL nor existing microarray products are amenable to a comparable high-throughput implementation at any cost.

## Conclusion

Genome-wide DNA microarray technology continues to be a mainstay of biologic discovery. However, the ability to measure gene expression signatures of interest with great rapidity and flexibility, and at very high throughput and dramatically lower cost, represents, in our view, the next transformative technology in the gene expression field. We believe that the LMF method described here has the potential for such impact.

## Materials and methods

### Cell culture and RNA isolation

HL60 (human promyelocytic leukemia) cells were cultured in RPMI supplemented with 10% fetal bovine serum and antibiotics. Cells were treated with 1 μmol/l tretinoin (all-*trans *retinoic acid; Sigma-Aldrich, St Louis, MO, USA) in dimethyl sulfoxide (DMSO; final concentration 0.1%) or DMSO alone for five days. Total RNA was isolated from bulk cultures with TRIzol Reagent (Invitrogen, Carlsbad, CA, USA), in accordance with the manufacturer's directions. For the classification exercise, microtiter plate cultures were treated with 200 nmol/l tretinoin or DMSO for two days to mimic the submaximal signatures likely to be encountered in a small molecule screen, and were and prepared for mRNA capture by the addition of Lysis Buffer (RNAture, Irvine, CA, USA).

### Microarrays

Total RNA was amplified and labeled using a modified Eberwine method, the resulting cRNA was hybridized to Affymetrix GeneChip HG-U133A oligonucleotide microarrays, and the arrays were scanned in accordance with the manufacturer's directions. Raw data were deposited in the National Center for Biotechnology Information (NCBI) Gene Expression Omnibus (GEO [13]) and are accessible through GEO series accession number GSE5007. Intensity values were scaled such that the overall fluorescence intensity of each microarray was equivalent. Expression values below an arbitrary baseline (20) were set to 20.

### Gene selection

The 9,466 probe sets reporting above baseline were first divided into upregulated and downregulated groups by differences in mean expression levels between tretinoin and vehicle treatments. Each of these groups was further divided into three sets of approximately equal size on the basis of the lower mean expression level. The selected basal expression categories were 20-60 (low), 60-125 (moderate), and >125 (high). Probe sets reporting small (1.5-2.5×), medium (3-4.5×), or large (>5×) changes in mean expression level within each basal expression category were extracted and ranked by signal to noise ratio. The top five probes mapping to unique RefSeq identifiers according to NetAffx [14] in each of the 18 categories were selected, populating nine sets of 10 genes (Additional data file 1).

### Probes and primers

Upstream probes were composed (5' to 3') of the complement of the T7 primer site (TAA TAC GAC TCA CTA TAG GG), a 24 nucleotide (nt) barcode, and a 20 nt gene-specific sequence. Downstream probes were 5'-phosphorylated, and contained a 20 nt gene-specific sequence and the T3 primer site (TCC CTT TAG TGA GGG TTA AT). Barcode sequences were developed by Tm Bioscience (Toronto, Ontarion, Canada) [15] and detailed in the Luminex FlexMAP Microspheres Product Information Sheet [8]. Gene-specific fragments of probes were designed against the Oligator Human Genome RefSet, keyed by RefSeq identifier, where available. A 40 nt region was manually selected from within these 70 nt sequences to yield two fragments of equal length with roughly similar base composition and juxtaposing nucleotides being C-G or G-C, where possible. Probe sequences are provided in Additional data file 2. Capture probes contained the complement of the barcode sequences and had 5'-amino modification and a C12 linker. The T7 primer (5'-TAA TAC GAC TCA CTA TAG GG-3') was 5'-biotinylated. The T3 primer has the sequence 5'-ATT AAC CCT CAC TAA AGG GA-3'. Oligonucleotides (all with standard desalting) were from Integrated DNA Technologies (Coralville, IA, USA).

### Beads and bead coupling

Luminex xMAP Multi-Analyte COOH Microspheres [8] were coupled to capture probes in a semi-automated microtiter plate format. Approximately 2.5 × 10^6 ^microspheres were dispensed to the wells of a V-bottomed microtiter plate, pelleted by centrifugation at 1800 *g *for 3 minutes, and the supernatant removed. Beads were resuspended in 25 μl binding buffer (0.1 M 2- [*N*-morpholino]ethansulfonic acid; pH 4.5) by sonication and pipeting, and 100 pmol capture probe was added. A volume of 2.5 μl of a freshly prepared 10 mg/ml aqueous solution of 1-ethyl-3-(3-dimethylaminopropyl) carbodiimide hydrochloride (Pierce, Milwaukee, WI, USA) was added, and the plate incubated at room temperature in the dark for 30 minutes. This addition and incubation step was repeated, and 180 μl 0.02% Tween-20 added with mixing. Beads were pelleted by centrifugation, as before, and washed sequentially in 180 μl 0.1% sodium dodecyl sulfate and 180 μl tris-EDTA (TE) (pH 8.0) with intervening spins. Coupled microspheres were resuspended in 50 μl TE (pH 8.0) and stored in the dark at 4°C for up to one month. Bead mixes were freshly prepared and contained about 1.5 × 10^5^/ml of each microsphere in 1.5× TMAC buffer (4.5 mol/l tetramethylammonium chloride, 0.15% *N*-lauryl sarcosine, 75 mmol/l tris-HCl [pH 8.0], and 6 mmol/l EDTA [pH 8.0]). The mapping of bead number to capture probe sequence is provided in Additional data file 3.

### Ligation-mediated amplification

Transcripts were captured in oligo-dT coated 384 well plates (GenePlateHT; RNAture) from total RNA (500 ng) in Lysis Buffer (RNAture) or whole cell lysates (20 μl). Plates were covered and centrifuged at 500 *g *for one minute, and incubated at room temperature for one hour. Unbound material was removed by inverting the plate onto an absorbent towel and spinning as before. A volume of 5 μl of an M-MLV reverse transcriptase reaction mix (Promega, Madison, WI, USA) containing 125 μmol/l of each dNTP (Invitrogen) was added. The plate was covered, spun as before, and incubated at 37°C for 90 minutes. Wells were emptied by centrifugation, as before. A volume of 10 fmol of each probe was added in 1× *Taq *Ligase Buffer (New England Biolabs, Ipswich, Ma, USA; 5 μl), the plate covered, spun as before, heated at 95°C for two minutes and maintained at 50°C for six hours. Unannealed probes were removed by centrifugation, as before. A volume of 5 μl of 1× *Taq *Ligase Buffer containing 2.5 U *Taq *DNA ligase (New England Biolabs) was added, the plate covered, spun as before, and incubated at 45°C for one hour followed by 65°C for 10 minutes. Wells were emptied by centrifugation, as before. A volume of 15 μl of a HotStarTaq DNA Polymerase mix (Qiagen, hilden, Germany) containing 16 μmol/l of each dNTP (Invitrogen) and 100 nmol/l of T3 primer and biotinylated T7 primer was added. The plate was covered, spun as before, and polymerase chain reaction performed in a Thermo Electron (Milford, MA, USA) MBS 384 Satellite Thermal Cycler (initial denaturation of 92°C for 9 minutes, 92°C for 30 s, 60°C for 30 s, 72°C for 30 s for 39 cycles; final extension at 72°C for 5 minutes). Total time from the addition of lysis buffer to hybridization-ready product for 96 samples processed in parallel in a single microtiter plate is approximately 14 hours.

### Hybridization and detection

A volume of 15 μl of LMA reaction product was mixed with 5 μl TE (pH 8.0) and 30 μl bead mix (about 4,500 of each microsphere) in the wells of a Thermowell P microtiter plate (Costar, Corning, NY, USA). The plate was covered and incubated at 95°C for two minutes and maintained at 45°C for 60 minutes. A volume of 20 μl of a reporter mix containing 10 ng/μl streptavidin R-phycoerythrin conjugate (Molecular Probes, Eugene, OR, USA) in 1× TMAC buffer (3 mol/l tetramethylammonium chloride, 0.1% *N*-lauryl sarcosine, 50 mmol/l tris-HCl [pH 8.0], 4 mmol/l EDTA [pH 8.0]) was added with mixing and incubation continued at 45°C for five minutes. Beads were analyzed with a Luminex 100 instrument [8]. Sample volume was set at 50 μl and flow rate was 60 μl/minute. A minimum of 100 events were recorded for each bead set and median fluorescence intensities (MFIs) computed. Total time from the start of hybridization to download of raw data from the instrument for 96 samples processed in parallel in a single microtiter plate is approximately three hours. Expression values for each transcript were corrected for background signal by subtracting the MFI of corresponding bead sets from blank (TE only) wells. Values below an arbitrary baseline (5) were set to 5, and all were normalized against an internal control feature (GAPDH_3).

### k-Nearest-neighbor classifier

The microarray-derived expression signature from long duration, high-dose tretinoin or vehicle treatments was used to train a series of KNN classifiers in the spaces of the full 90-member gene set and each of the nine 10-member gene categories. These were applied to the corresponding data from the 88 LMF test samples whose internal reference feature (GAPDH_3) was within two standard deviations from the mean. To permit the cross-platform analysis, both the train and test data sets were normalized so that each gene had a mean of zero and a standard deviation of one. The KNN algorithm classifies a sample by assigning it the label most frequently represented among the *k *nearest samples. In this case *k *was set to 3. The votes of the nearest neighbors were weighted by one minus the cosine distance. This analysis was performed with the GenePattern software package [16].

## Additional data files

The following additional data are included with the online version of this article: An Excel file listing the genes populating the representative gene space (Additional data file 1); an Excel file containing the LMF probe sequences (Additional data file 2); an Excel file providing a mapping of bead number to capture probe sequence (Additional data file 3); a document containing the Affymetrix data used to select the representative gene space (in the tab-delimited .res file format; Additional File 4); a document containing raw LMF data used to assess the reproducibility of the method and for comparisons between platforms (in the tab-delimited .txt file format; Additional data file 5); a document containing raw LMF data used to assess the stability of the LMF method (in the tab-delimited .txt file format; Additional data file 6); a document containing raw LMF data used to assess the stability of the LMF method (in the tab-delimited .txt file format; Additional data file 7); and a document containing raw LMF data from the analysis of the microtiter cultures, which were used in the classification exercise (in the tab-delimited .txt file format; Additional data file 8).

## Supplementary Material

Additional data file 1 The genes populating the representative gene space Click here for file

Additional data file 2 The LMF probe sequences Click here for file

Additional data file 3 A mapping of bead number to capture probe sequence Click here for file

Additional data file 4 A document containing the Affymetrix data used to select the representative gene space Click here for file

Additional data file 5 A document containing raw LMF data used to assess the reproducibility of the method and for comparisons between platforms Click here for file

Additional data file 6 A document containing raw LMF data used to assess the stability of the LMF method Click here for file

Additional data file 7 A document containing raw LMF data used to assess the stability of the LMF method Click here for file

Additional data file 8 A document containing raw LMF data from the analysis of the microtiter cultures, which were used in the classification exercise Click here for file

## Figures and Tables

**Table 1:** Classification accuracy

		Differential expression		
		1.5-2.5×	3-4.5×	>5×
Basal expression level	20-60	87.5	97.7	97.7
	60-125	85.2	98.9	94.3
	>125	98.9	98.9	100

Accuracy (%) of a *k*-nearest-neighbor classifier trained on microarray data to predict the true identity (tretinoin or dimethyl sulfoxide) of 88 test samples in the space of each of the nine gene classes from Figure 3.
